# microUSV: A low-cost platform for indoor marine swarm robotics research

**DOI:** 10.1016/j.ohx.2020.e00105

**Published:** 2020-03-21

**Authors:** Calvin Gregory, Andrew Vardy

**Affiliations:** a Department of Ocean and Naval Architectural Engineering, Memorial University of Newfoundland, Canada; b Department of Computer Science and Department of Electrical and Computer Engineering, Memorial University of Newfoundland, Canada

**Keywords:** Unmanned surface vehicle, Autonomous surface vehicle, Boat, Swarm, Multi-robot system

## Abstract

This article describes an open source Unmanned Surface Vehicle (USV) designed to operate in indoor laboratory environments. The microUSV is small (23 cm long) and inexpensive (approximately $320 per unit for 10 vessels): an ideal hardware platform for algorithm validation and marine swarm robotics research. The primary design goal was to minimize the vehicle’s size and cost while providing a stable and maneuverable platform with onboard autonomy. To that end the vehicle is built using 3D printed and off-the-shelf hobbyist electronic components and uses an overhead camera system to simulate sensor data to minimize the number of onboard sensors required. This article describes the context, design, and assembly procedures for a microUSV and demonstrates the platform’s base-level functionality in the form of a waypoint following controller implementation for both single and multi-robot configurations.

## Graphical abstract

**Figure:**
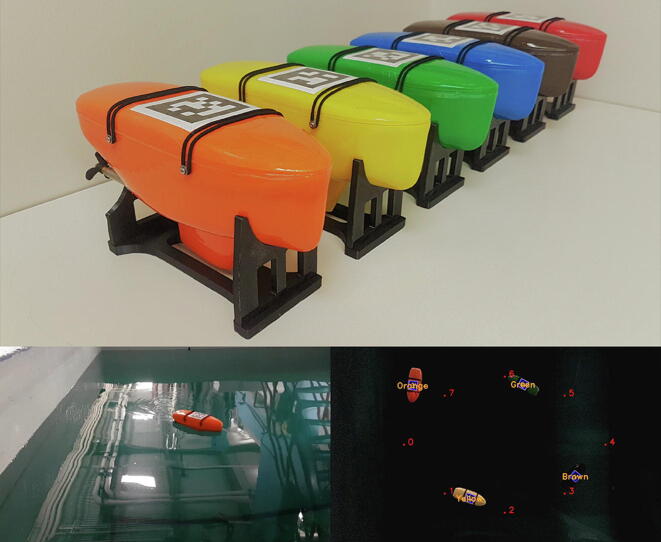

**Specifications table:**

**Table:** 

Hardware name	microUSV
Subject area	Engineering and Material Science
Hardware type	Electrical engineering and computer science
Open source license	Hardware: CERN Open Hardware License v1.2
	Software: GNU General Public License v3.0
Cost of hardware	$522 CAD per vessel (single vessel), $317 CAD per vessel (10 vessels)
Source file repository	https://doi.org/10.17605/OSF.IO/7FQ6U

## 1 Hardware in context

Unmanned Surface Vehicles (USV) are autonomous boats capable of augmenting or even replacing the use of manned vessels in dangerous and/or monotonous tasks. These platforms are commonly used for oceanographic research and military applications such as surveillance and environmental monitoring [1]. Designed to survive in harsh open-water environments, these vehicles are typically large and expensive. The majority of USVs are custom designed and built for their target application and are not generally available for purchase commercially. Those that are available on the market can range in size from 1.3 to 8 meters in length. These commercial vessels can cost between $30,000 and several million USD depending on their sensor payload [2], [3], [4]. This makes the cost of purchasing a fleet of commercially available USVs prohibitive for many laboratories and researchers.

We are investigating the use of multi-robot systems as a means of environmental monitoring and cleanup as part of our ongoing research. The intended application for our proposed platform is to test algorithms developed for swarm robotics, such as the approach described in [5], as methods for ocean contaminant collection using a fleet of USVs.

As the name implies, multi-robot systems require multiple robots. Simulated environments can allow multiple robots to be easily generated and tested but to truly capture the complexity of the marine environment for algorithm validation, hardware testing is also necessary [6]. The high cost associated with purchasing multiple commercial USVs motivated the search for a low-cost alternative platform.

Open source USV platforms offer an alternative to purchasing commercial options. Open source solutions are generally much less expensive and can be built and modified by an individual user or researcher to suit their specific needs. Such platforms are slowly being introduced to the research community such as the Jetyak [7], ARCAB [8], and SMARTBoat 3 [9]. These three platforms have approached the problem of low-cost USV research platforms from different angles.

The Jetyak is a fully functional USV capable of carrying the same instrumentation and navigation equipment as large commercial USVs, however its $15,000 USD fabrication cost, while cheaper than commercial USV systems, make it too expensive to produce in large quantities for multi-robot systems research. The Arctic Research Centre Autonomous Boat (ARCAB) has the same issue as the Jetyak for our application: at 3000 euro per vessel the cost of acquiring multiple vessels is prohibitive. The SMARTBoat 3 is a recently published system with a much lower reported cost of $200 per vessel. Designed as a small environmental monitoring vessel, its shape was constrained by the use of repurposed off-the-shelf components for floatation. The SMARTBoat 3’s toroidal hull-form does not reflect the dynamics of larger USV’s, the eventual target platform for any algorithms developed through our experiments. This makes it an unsuitable platform for our purposes.

The size and payloads of full-scale USVs require outdoor testing environments whose availability can depend on weather and logistical factors. Ideally there would be an intermediary hardware testing platform capable of operating in a laboratory environment such as the system described in [10]. Such a testbed could be used as a stepping-stone to evaluate algorithms before investing in larger, more costly hardware for open water experiments on lakes or oceans.

To be considered a USV, a marine vessel must be capable of three things: locomotion, localization, and some degree of automatic control. Locomotion is a prerequisite for any vehicle, as vehicles must be capable of moving to be considered as such. To be considered an unmanned vehicle, there must be no human operator(s) aboard the vessel. With no operator aboard, a USV must have partial, if not full, automatic control. In robotics, this will often take the form of an onboard computer or microprocessor reacting to measurements from the vessel’s sensor. One or more of these sensors must provide some means of localization to produce the feedback measurements needed for the onboard controller. To the best of our knowledge, no such platform exists that is available for purchase commercially. Those indoor platforms that do exist are typically custom built for a given researcher or research group.

A remote control (RC) boat would fit the definition of a USV however such platforms require human-in-the-loop control and lack other desirable properties for marine robotics research such as onboard processing. An RC boat could be modified to include an onboard controller and remove the need for human intervention; In fact, similar things have been done successfully by hobbyists [11], [12], [13]. Such hobby projects served as a jumping-off point for our own solution.

**Fig. 1:**
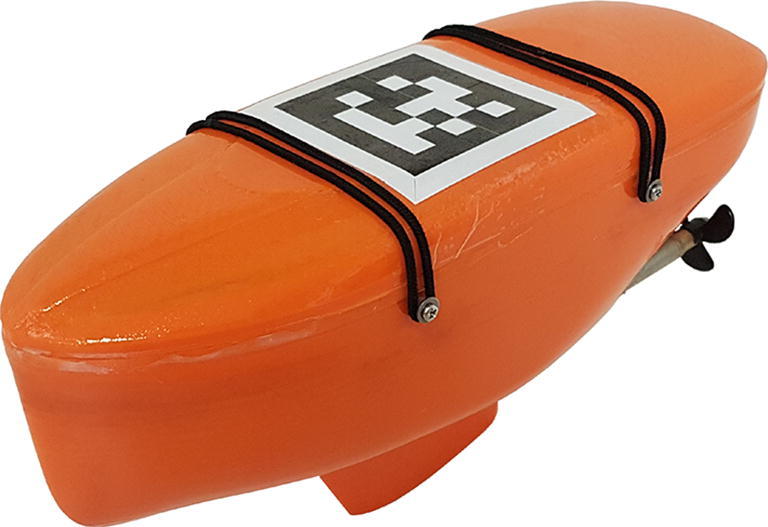
microUSV.

Finding no available USV platforms suitable for swarm robotics research, we have developed our own: The microUSV, shown in Fig. 1, is a small USV platform designed for use in laboratory environments. It was designed to be inexpensive to produce, capable of onboard autonomous control, and easily reprogrammable. The design leverages 3D printing and off-the-shelf hobbyist electronics to produce a marine robotics research platform that costs just over $500 CAD for a single unit and is much cheaper when produced in multiples. It measures 23 cm in length and so is capable of maneuvering in the 3.65 m × 3.65 m enclosed indoor water tank available to us for testing that would barely fit the smallest commercial USV platforms. A fleet of such vessels can be used to test and validate new or existing multi-robot systems applications in the marine environment such as object aggregation or dispersion, flocking behaviors, and collective mapping tasks.

This article, coupled with the project repository Wiki [14], will describe the procedures to assemble and test a microUSV and demonstrates the platform’s basic functionality.

## 2 Hardware description

### 2.1 Mechanical systems

The microUSV’s hardware can be broadly divided into three subassemblies: The hull, propulsion system, and onboard electronics bracket.

#### 2.1.1 Hull

**Fig. 2:**
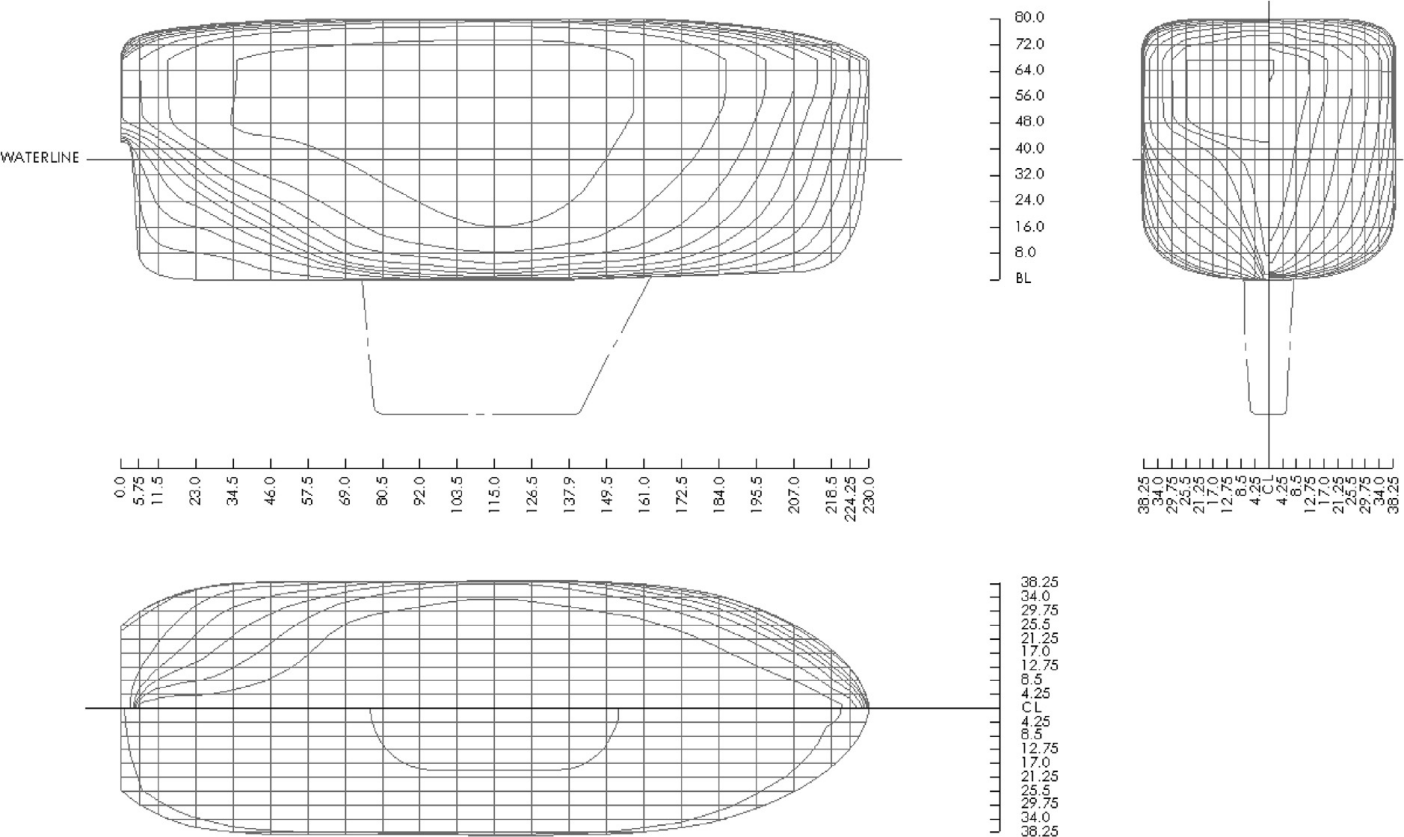
microUSV Hull Lines Plan (Dimensions in mm).

The first notable aspect of the microUSV’s hull design is its size: the vessel has a length overall of 230 mm, a beam of 89.2 mm, and a depth of 121.5 mm as seen in Fig. 2. A laboratory tank is a tightly confined operating environment when compared to a lake or ocean, with a surface area measured in m^2^ rather than km^2^. A small vessel is better suited to operating in such a small body of water than a full sized USV designed for the open water. A full sized USV with a length overall of 1.3 m, such as the vessel described in [2], or greater would be nearly incapable of maneuvering in a 3.65 m × 3.65 m test tank. One or more scaled-down vessels are fully capable of maneuvering in such a confined environment and, by extension, are better at demonstrating the behaviors being investigated by the operator(s).

A small vessel design is also beneficial for its reduced material costs: A smaller vessel requires smaller parts which influences not only the volume of materials required but also their selection. Using smaller parts reduces the vessel’s weight which results in lower loads on the hull so structural components can be made smaller and lighter. The microUSV’s hull design eschews more traditional structural materials like steel and aluminum for parts made of polylactic acid (PLA) plastic produced using an Ultimaker 2+ 3D printer. Multiple plastic hull designs were prototyped, and the material was deemed strong enough to support the vessel’s small operating loads.

The vessel’s hull form was designed using the lines plan of a tugboat [15] for reference. A tugboat hull was chosen for its stability and compact design. Good stability is of particular importance to the microUSV due to the use of an external pose detector system which will be discussed in Section 2.3. The compactness of this hull type is beneficial due to the increased usable internal cavity volume relative to the vessel’s size. The vessel’s low length-to-beam ratio is important to allow sufficient width of the internal volume for onboard electronics to be mounted without greatly increasing the vessel’s length overall. The microUSV’s hull was further compressed lengthwise relative to the reference lines plan to decrease this length-to-beam ratio and allow the hull to be fabricated as a single component on the 3D printer’s limited build platform space.

#### 2.1.2 Stability

The importance of mounting onboard electronics also influenced the decision to use a monohulled design over the catamaran design often seen in other small USVs [2], [4]: Mounting heavy components like batteries at or below the waterline improves the vessel’s stability without increasing the beam length which results in a vessel with a smaller footprint, better able to navigate confined spaces. Due to the microUSV’s small size, a catamaran design would require the twin hulls to be scaled up to provide enough internal space for component mounting. They would quickly exceed the volume necessary to keep the vessel afloat and further increase the vessel’s footprint. Since the microUSV operates exclusively indoors it does not need to survive wind and large waves, so the catamaran’s tradeoff in size for stability was deemed unnecessary. The microUSV’s monohulled design utilizes other means to achieve the necessary stability.

A fin keel was added to improve vessel stability without resorting to widening the hull. The keel’s surface area catches water as the vessel rolls, inducing a drag force to resist the motion and a steel ballast plate in the keel lowers the vessel’s center of gravity, thus increasing the metacentric height (GM). Molland [16] defines GM as ‘The vertical separation of the metacenter and the center of gravity as projected on to a transverse plane’ where the metacenter is ‘The intersection of successive vertical lines through the center of buoyancy as a ship is heeled progressively’, heel angle referring to the degree of roll a ship is experiencing. The metacenter is the effective pivot point for a ship experiencing roll and its height dictates the stability of a vessel.

**Fig. 3:**
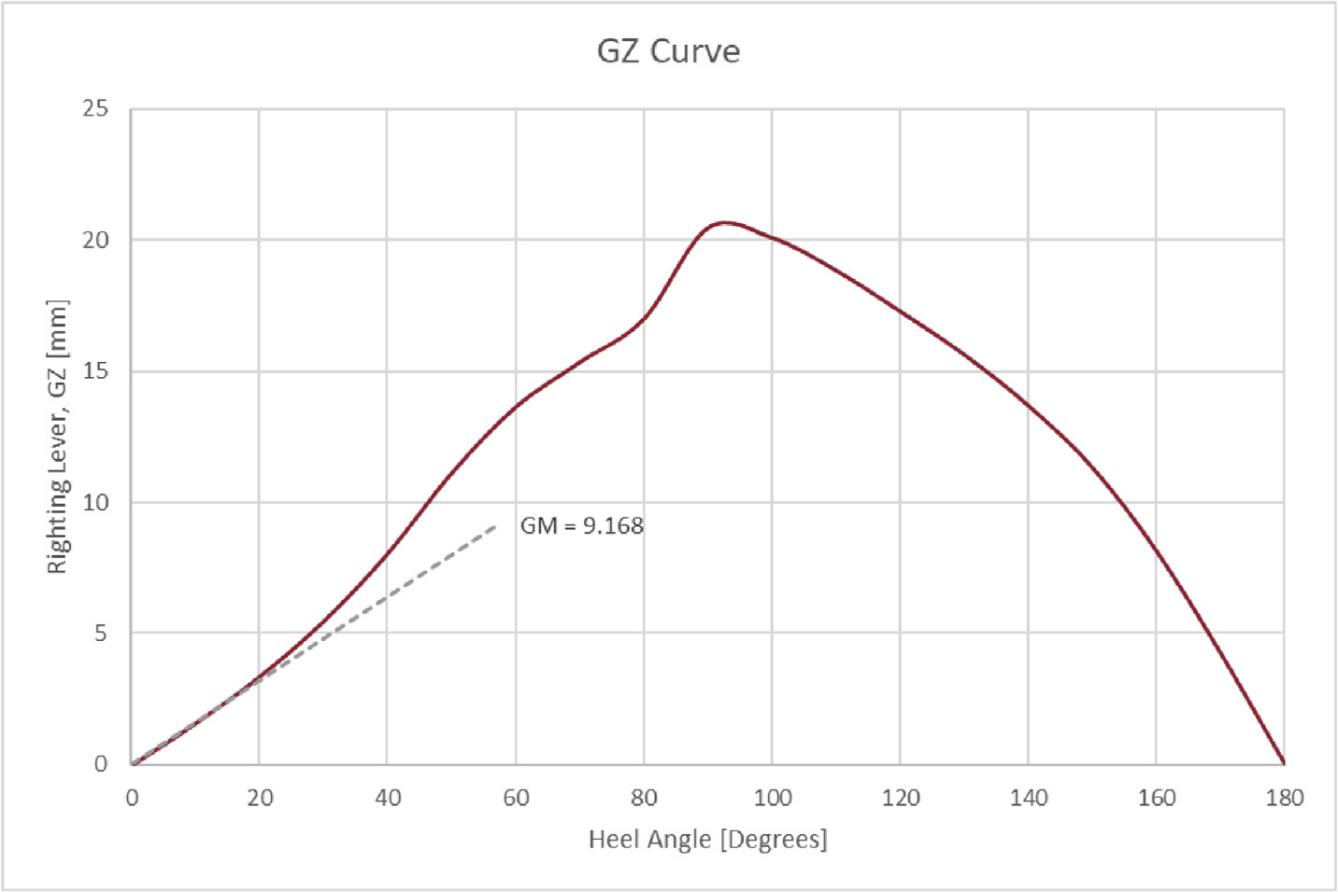
Righting Lever Length vs Heel (Roll) Angle Plot.

Increasing a vessel’s GM increases its stability due to the resultant increase in the vessel’s righting lever (GZ). GZ is the horizontal distance between a vessel’s center of gravity and the line of action of its buoyancy force. When offset due to roll, the gravity and buoyancy forces generate a couple moment which rights the vessel. A longer righting lever increases the righting moment experienced by a vessel as it rolls, returning it to an upright position faster. In the case of the microUSV, the vessel has an estimated natural roll period of 1.17 s [17]. The microUSV’s GZ curve, calculated using an iterative righting lever simulation with respect to vessel heel angle [18] and shown in Fig. 3, shows no angle of vanishing stability, defined as the x-intercept on a vessel’s GZ curve, meaning it can theoretically right itself from any degree of roll.

#### 2.1.3 Waterproofing

The hull design includes a lid to keep water out of the internal cavity in the case of excessive roll or splashes. This lid is mounted to the hull using a double lap joint around the hull’s entire upper edge and is kept in place by a pair of hair elastics. The design does not include a gasket or O-ring and so is splash proof but not completely watertight. In order to achieve a fully waterproof design a gasket flange or O-ring groove would need to be added with many tightly spaced bolts along the joint between hull and lid to squeeze the sealing elastomer component. These bolts would add significant weight to the vessel, requiring increased buoyancy, and therefore hull size, to support them and would shift the center of gravity upward, reducing the vessel’s GM and stability. The microUSV was designed to operate on the water’s surface and should never need to endure being submerged under water, so the costs of a fully waterproof design outweighed its benefits.

The vessel has, however, been successfully tested for very brief periods of immersion: it can survive for one second fully submerged in water without any water reaching the internal cavity and can likely endure longer immersions, but this has not been tested. It is easily capable of surviving the splash from a wave or an accidental immersion during retrieval.

The PLA plastic used for the hull is naturally waterproof, but it is also a biodegradable material: Its integrity may gradually deteriorate due to exposure to UV light or temperature changes over time. Additionally, the layered building process used by fused deposition modeling (FDM) 3D printers is imperfect. There is a chance of introducing small, often unnoticeable, defects and gaps in the walls of a part during the printing process. Although a 3D printed hull made with an accurate printer can easily survive a handful of immersions in water, it is unlikely to endure months or years of repeated use without leaking which would likely destroy the electronics housed inside. Therefore, an additional waterproof coating of epoxy was added to the hull’s external surfaces.

#### 2.1.4 Propulsion system

**Fig. 4:**
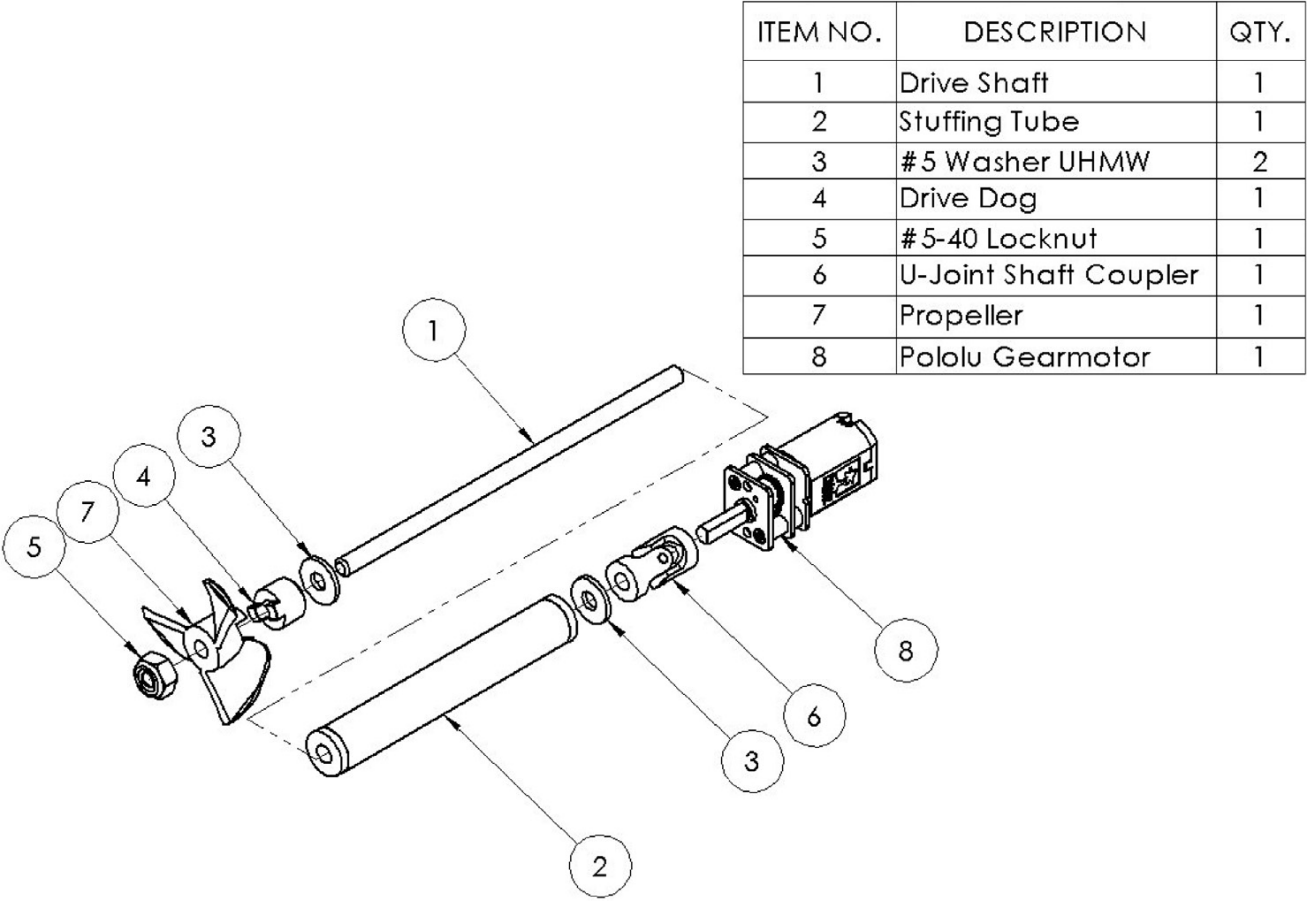
Drive Train Assembly Exploded View Drawing.

The microUSV’s propulsion system consists of a pair of propellers driven directly by DC motors, the components for which can be seen in Fig. 4. Each motor connects to its drive shaft via a universal joint shaft coupler to mitigate small shaft misalignments. The drive shafts are arranged in parallel with a spacing of 48 mm and independently controlled motors to allow differential drive. A differential drive system was chosen over a more traditional propeller and rudder arrangement to improve the vessel’s maneuverability: making the microUSV better able to navigate its confined operating environment.

The vessel’s drive shafts are kept watertight with a pair of custom stuffing tubes. A stuffing tube is a simple assembly packed with grease which forms a seal around a rotating shaft. The microUSV’s stuffing tubes are made of stainless steel with a bushing at each end to support the shaft as it rotates and the void space between shaft and tube is filled with petroleum jelly. These tubes are mounted to the hull and themselves sealed in place using a marine-grade silicone sealant. The stuffing tubes needed to be custom made as all existing off-the-shelf stuffing tube and drive shaft systems were too long to fit the vessel’s hull.

The propulsion system consists of mostly off-the-shelf components to reduce costs: the propellers, drive dogs, and shaft couplers were all sourced from RC boat part suppliers. The drive shaft, motors, and components for the stuffing tubes were also purchased off-the-shelf but required further modification.

#### 2.1.5 Onboard electronics bracket

**Fig. 5:**
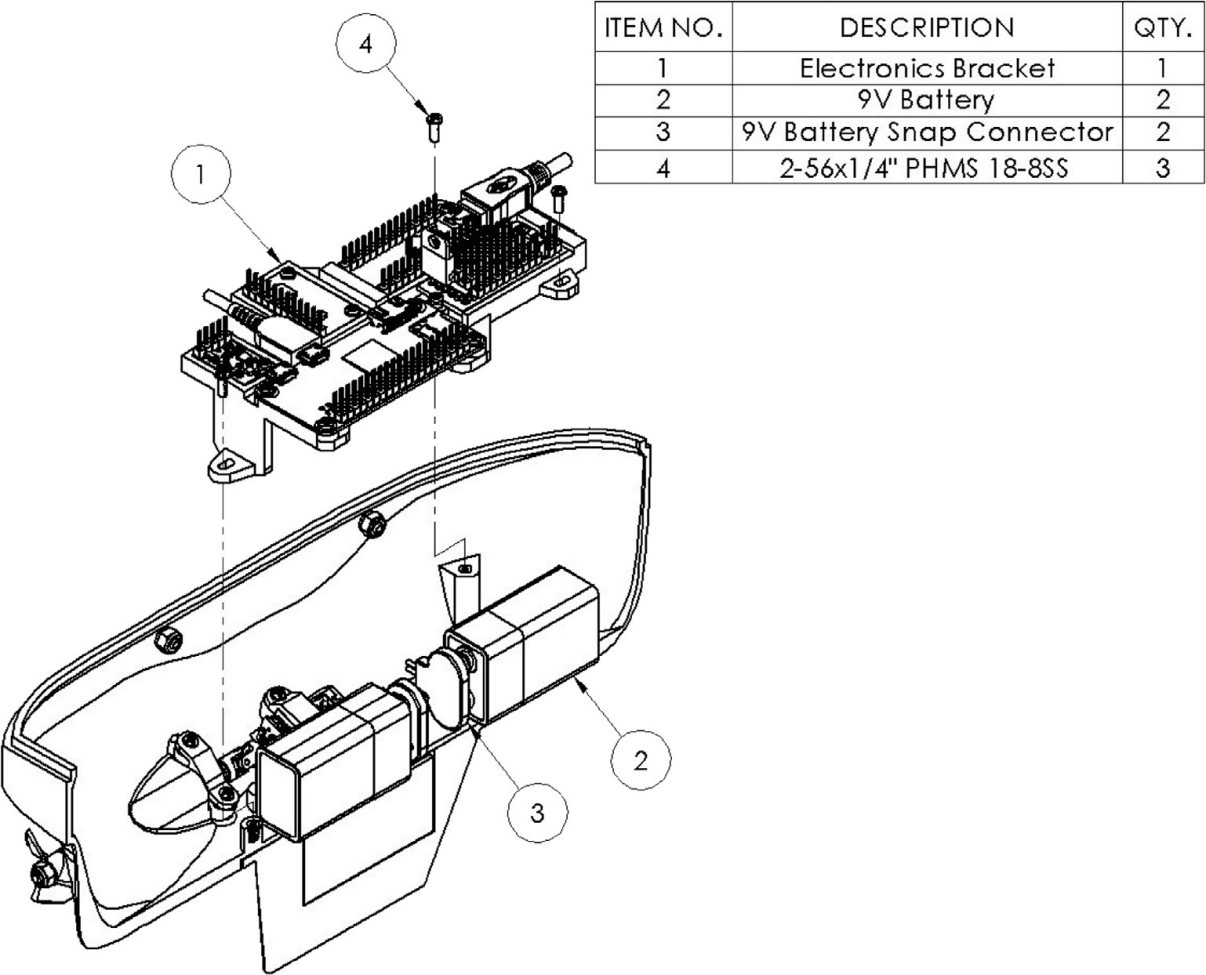
Electronics Bracket Assembly Drawing.

The electrical components onboard the microUSV are all mounted to a 3D printed bracket except for the batteries and motors which are mounted directly to the hull. The motors are mounted independently to ensure alignment with the drive shafts and the batteries are mounted below the electronics bracket in order to keep the vessel’s center of gravity as low as possible to improve stability as discussed in Section 2.1.2, Stability. This electronics bracket is mounted on three threaded posts inside the hull and can be easily removed to change batteries or bench test and debug the electrical system. The mounting process is illustrated in Fig. 5.

### 2.2 Electrical system

**Fig. 6:**
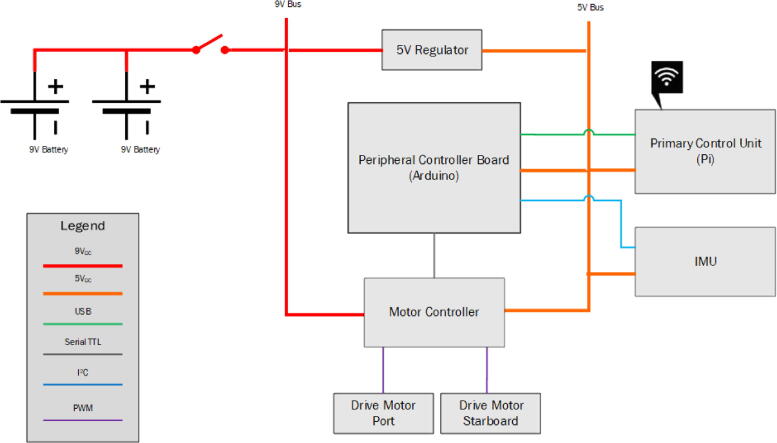
System Integration Diagram.

The microUSV’s onboard electronics system is designed to utilize readily available hobbyist components. This minimizes cost and allows damaged or defective components to be replaced quickly. It also grants access to the large user communities supporting each of these products, potentially simplifying the debugging process for a new user. A system integration diagram is provided in Fig. 6.

#### 2.2.1 Electronic devices

To achieve onboard autonomous control and reprogrammability, a single-board computer was selected as the primary control unit: A Raspberry Pi Zero W. The Pi Zero is the smallest of the popular family of pocket-sized computers and is well suited to small robotics projects. The Pi Zero W features a built-in wireless module used for communication between each vessel and the host PC discussed in Section 2.3. It runs the Raspbian operating system which grants it the same functionality as most desktop Linux systems. This allows the microUSV’s onboard control software to be written in any number of popular high-level programming languages and easily changed during testing.

The Raspberry Pi communicates with an Arduino Nano, which serves as a peripheral control device. The Arduino is included to simplify the interface between the vessel’s primary controller and peripheral devices; a motor controller and inertial measurement unit (IMU). It also offers room for expansion with eight unused digital pins and six unused analog pins available for additional devices or sensors.

**Fig. 7:**
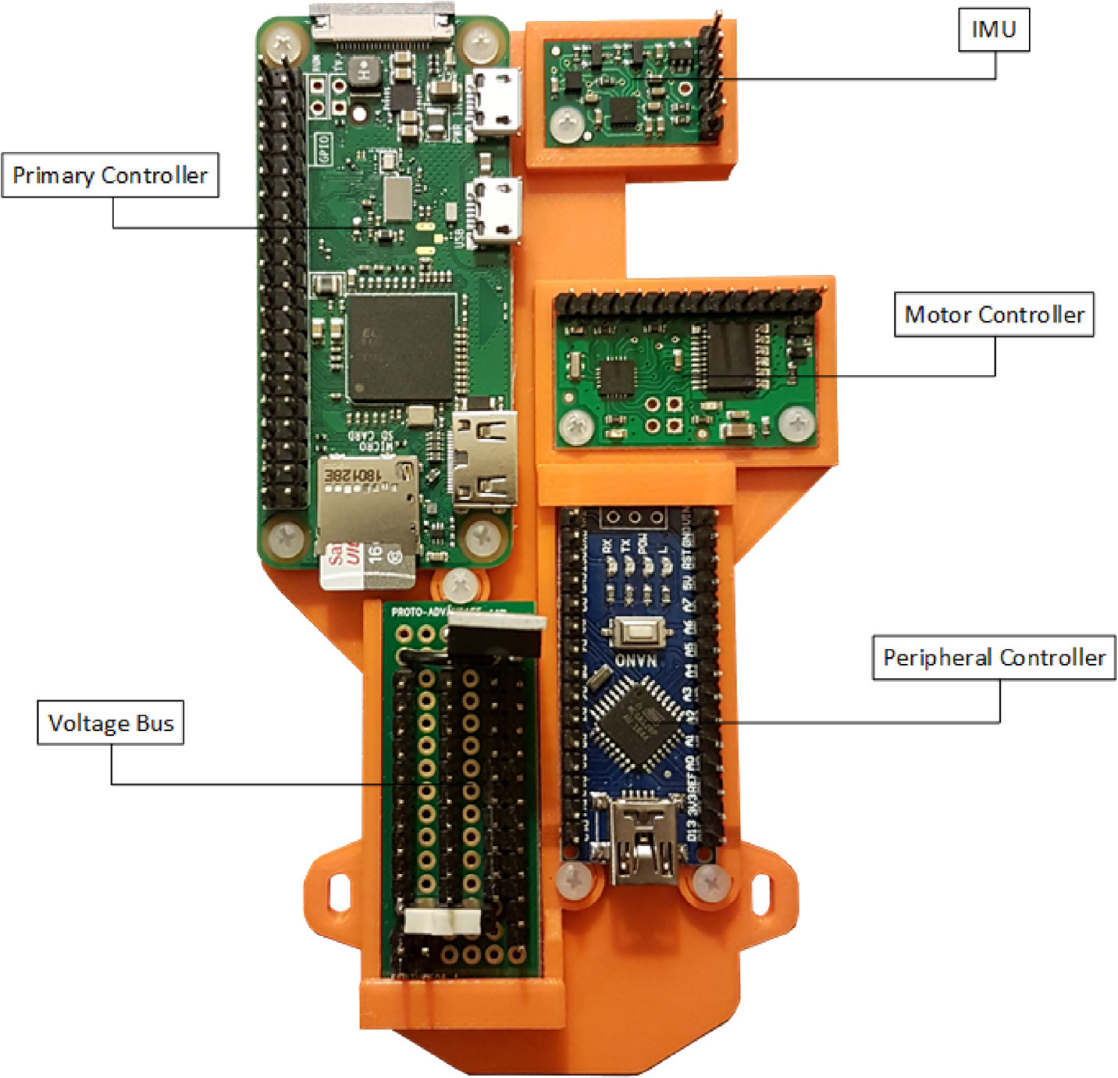
Electronics Bracket with Labelled Components.

The peripheral devices, a Qik 2s9v1 dual serial motor controller and MinIMU-9 v5, were both sourced from Pololu Robotics and Electronics. These are each very small devices with easily accessible drivers and support communities which fulfill their designed rolls in the microUSV system: The IMU is included to augment any custom odometry system a user may deploy while the motor controller drives the vessel’s motors. All onboard electronic devices are shown in Fig. 7.

The motors selected for the microUSV were also sourced from Pololu. They are tiny 12 V DC motors with a 5:1 gearbox. Since the torque requirements for the chosen 28 mm diameter propeller were so small, the priorities when selecting a motor were its small size and power consumption. The motor’s voltage rating was also a primary consideration to interface with the microUSV’s power system.

#### 2.2.2 Power system

The vessel’s power system was designed to be as simple as possible. Power is provided by a pair of standard 9 V alkaline batteries. Using standard batteries greatly simplifies vessel maintenance and they are much cheaper than lithium-polymer batteries which are popular in mobile robotics applications. Since the microUSV operates exclusively indoors, ease of maintenance was deemed more important than extended battery life as the vessels can be easily pulled from the water and have fresh batteries installed between experiments if necessary.

**Fig. 8:**
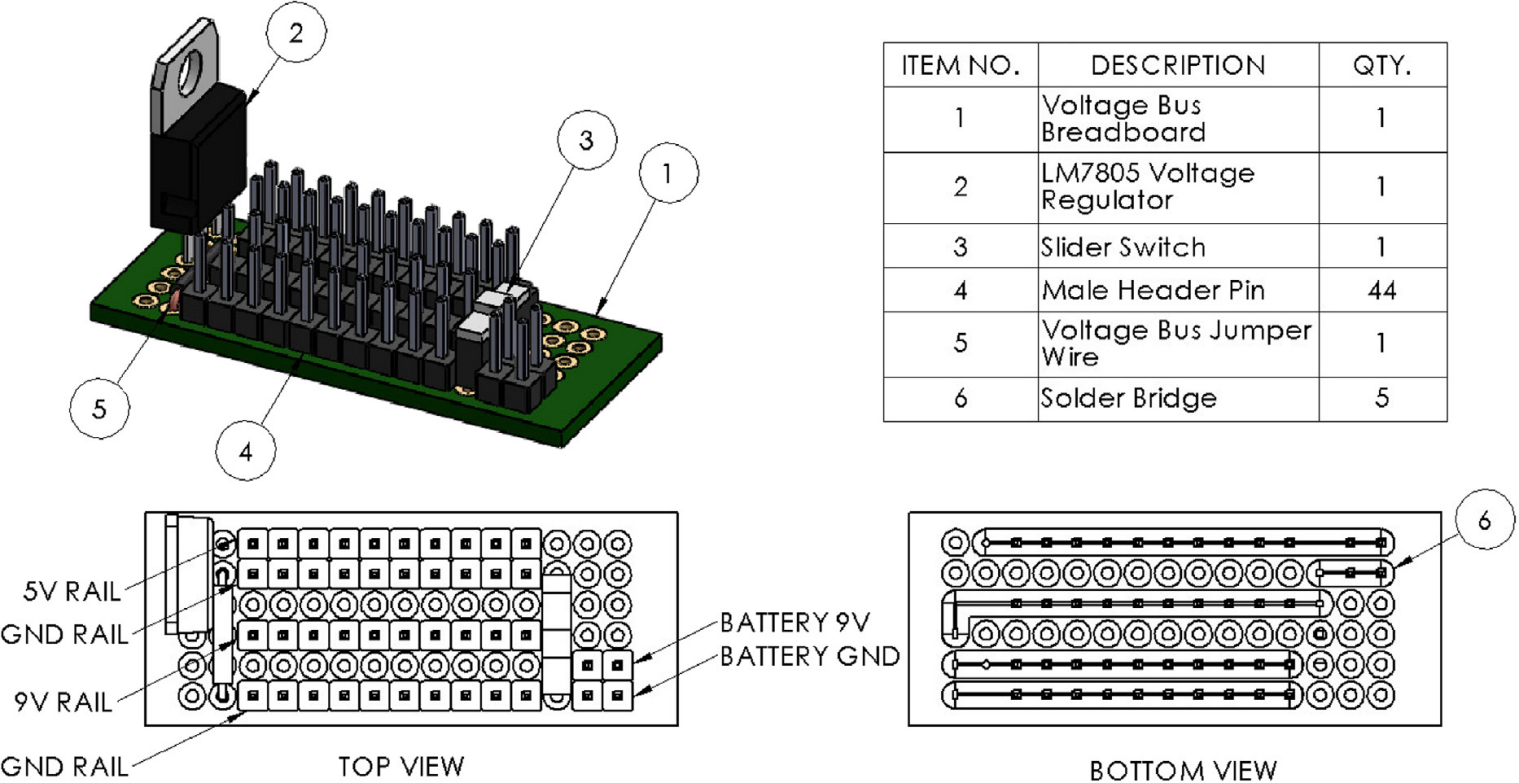
Voltage Bus Drawing.

The Raspberry Pi, IMU, and motor controller cannot accept the 9 V battery power with a maximum input voltage for each device ranging from 5 V to 5.5 V. A simple voltage regulator circuit, shown in Fig. 8, was added to the design to accommodate these requirements providing regulated 5 V DC and unregulated 9 V DC. The unregulated 9 V DC power is used to drive the motors. This circuit doubles as a voltage bus allowing devices to be quickly connected to power without disturbing the rest of the system using standard 2.54 mm header pin connectors. The 5 V section of the voltage bus has its ground and live rail running adjacent to each other while the 9 V section has a 2.54 mm (one pin row) separation between its live and ground rails. By using the appropriately sized connector housings on their jumper wires, a two-pin housing for 5 V devices and a 3-pin housing with a gap in the middle slot for 9 V devices, the risk of connecting a device to the wrong input voltage is greatly reduced.

### 2.3 Control software

The sensors usable on the microUSV are limited due to the vessel’s size and indoor operating environment. Traditional navigation sensors for full scale USVs such as GPS and magnetometer cannot function in indoor environments and powerful rangefinder systems such as LIDAR can weigh as much as the rest of the microUSV’s components combined and needs to be mounted high on the vessel to function properly. Mounting one of these systems onboard would quickly sink or flip the vessel. Those sensors that are small and light enough to fit the microUSV cannot produce data of sufficient quality to be reliable. Unable to use traditional odometry sensors, we have opted to use an external pose detector system instead. Similar to the Augmented Reality for Kilobots system [19], the addition of virtual sensors using external hardware allows for more complex behaviors without greatly increasing the hardware cost or complexity of the vessels.

Each microUSV is marked with a unique AprilTag attached to its lid. These tags allow the position of each vessel to be tracked when in view of an overhead camera using the AprilTag detection algorithm [20]. These tags must be kept in view of the camera in order to receive up-to-date pose. The AprilTag detection algorithm can detect tags with a large degree of skew but to ensure the most reliable performance the microUSV design attempts to minimize roll motion and so must be very stable, a dominant factor in the vessel’s hull design.

#### 2.3.1 Peripheral devices

**Fig. 9:**
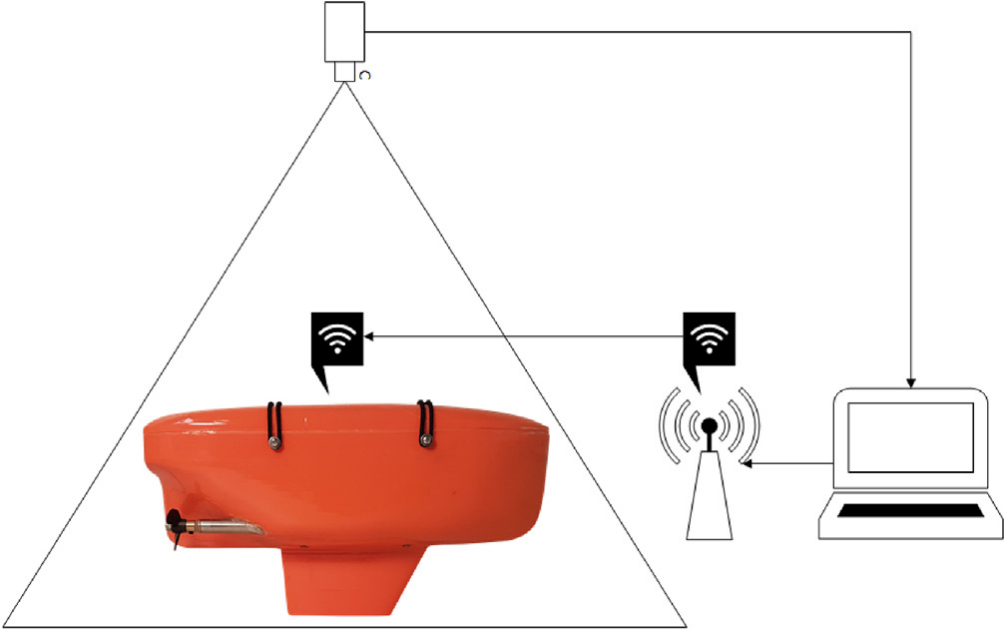
External Pose Detector System Data Flow Diagram.

In addition to the vessel’s themselves, several other devices are required for the microUSV system to function: A USB camera, a computer, and a wireless router. Fig. 9 illustrates the arrangement of these devices to form the external pose detector system.

A camera was chosen over a commercial pose tracking system due to the significantly lower cost. The camera, as mentioned previously, must be mounted above the operating area in order to capture video of the vessels or more specifically, their AprilTags. A Logitech C920 camera was used for this purpose during development. A higher video feed resolution allows for detections at a longer range, thus increasing the size of the operating area however the increased video bandwidth demands more processing time and so can introduce latency. The best results were achieved during testing using the camera set to output a 720p video feed. The camera is connected via USB to a computer, referred to henceforth as the host PC.

The host PC is responsible for running the AprilTag detection algorithm to continuously update a 2D pose estimate for each microUSV visible in the video feed. Our testing computer was running an Intel i7-8750H CPU. It also acts as a server, receiving update requests from the microUSVs and responding with simulated sensor data messages. The host PC sends these messages over a wireless network via a router to which all the microUSVs are connected. The wireless router used in our testing was a NETGEAR Nighthawk AC1900 however a router of this quality is not necessary. Each vessel requires approximately 200 Bytes/s of bandwidth so a small fleet of vessels can easily be managed by a low-end wireless router.

**Fig. 10:**
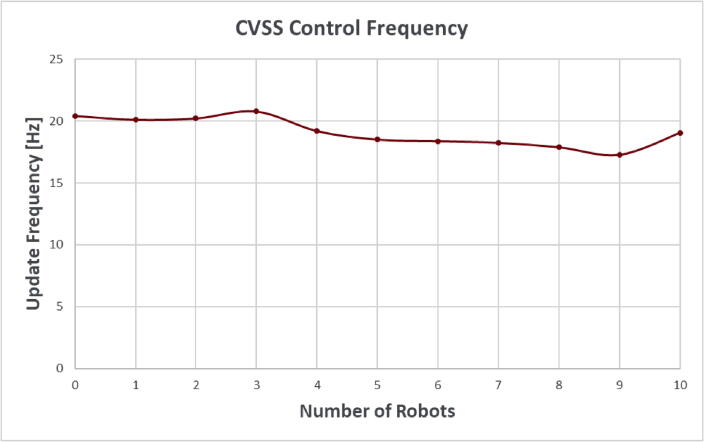
Host PC Software Update Frequency vs Number of Robots on the Network.

Fig. 10 shows that the host PC software is able to maintain a stable pose estimate update rate between 17 and 21 Hz while managing a fleet of up to 10 vessels.^1^ This update frequency is sufficient for simple navigation tasks even without utilizing the onboard IMU to estimate pose between updates.

#### 2.3.2 Host PC software

The host PC runs a C++ application called CVSensorSimulator: This application uses computer vision to continuously estimate the sensor values observed by each microUSV. Its primary purpose is providing a global pose estimate to each vessel using the AprilTag library [20] written in C. As a global manager agent, it has access to all pose estimates simultaneously and so can also be used to simulate other sensors for behaviors such as collision avoidance and target tracking. The perfect information available to the application allows it to simulate different inter-vessel communication schemes by artificially limiting access to the data of other vessels. This can allow a researcher to test algorithms using multiple communication schemes without modifying the hardware involved. As an example, a system with no inter-vessel communication can be simulated by restricting a vessel’s sensor data access to its own data. A system with limited inter-vessel communication range can be simulated by restricting a vessel’s sensor data access to its own data in addition to the data of any other vessels within an arbitrary distance.

Since USVs operate in an effectively planar environment, the application extracts each vessel’s 2D pose from its AprilTag detection: x position, y position, and heading angle (yaw). This pose is stored in a local object for each microUSV. A separate thread acts as a server awaiting update request messages. When a vessel requires updated sensor data, it sends a message to the host PC which then takes the most recent pose data from the local object representing that vessel and sends it back. These messages are constructed using Google’s Protocol Buffer library [21], a language-neutral tool used to serialize data structures that requires less data than more traditional solutions such as XML. This reduces the bandwidth requirement for the wireless router.

#### 2.3.3 Vessel software

There are two devices onboard each vessel running our custom software: A Raspberry Pi (the primary controller) and an Arduino (the peripheral controller). The Arduino application, called PeripheralController, simply acts as a pass-through device for motor speed messages. It receives integer motor speed commands from the primary controller and forwards them to the Qik motor controller using Pololu’s Arduino library for the device. This application can also be modified to include an interface to the onboard IMU, also a Pololu device with an accompanying library, which connects to the Arduino. The IMU data can be used to augment a state estimation system implemented on the Raspberry Pi.

Two applications have been written for the microUSV’s primary controller: MUSVController and Teleop. These applications were both written in Python due to the Raspbian operating system’s native support for the language and the ability to prototype quickly. The Teleop application allows a user to operate the microUSV remotely over Secure Shell (SSH) using their keyboard and is intended as a tuning and debugging tool. The MUSVController application contains the logic for autonomous control. The MUSVController application implements a simple waypoint following algorithm: The controller manages its distance-to-target using a proportional controller and a PI controller to handle its heading angle. New users are encouraged to augment or replace this controller for their own work.

**Fig. 11:**
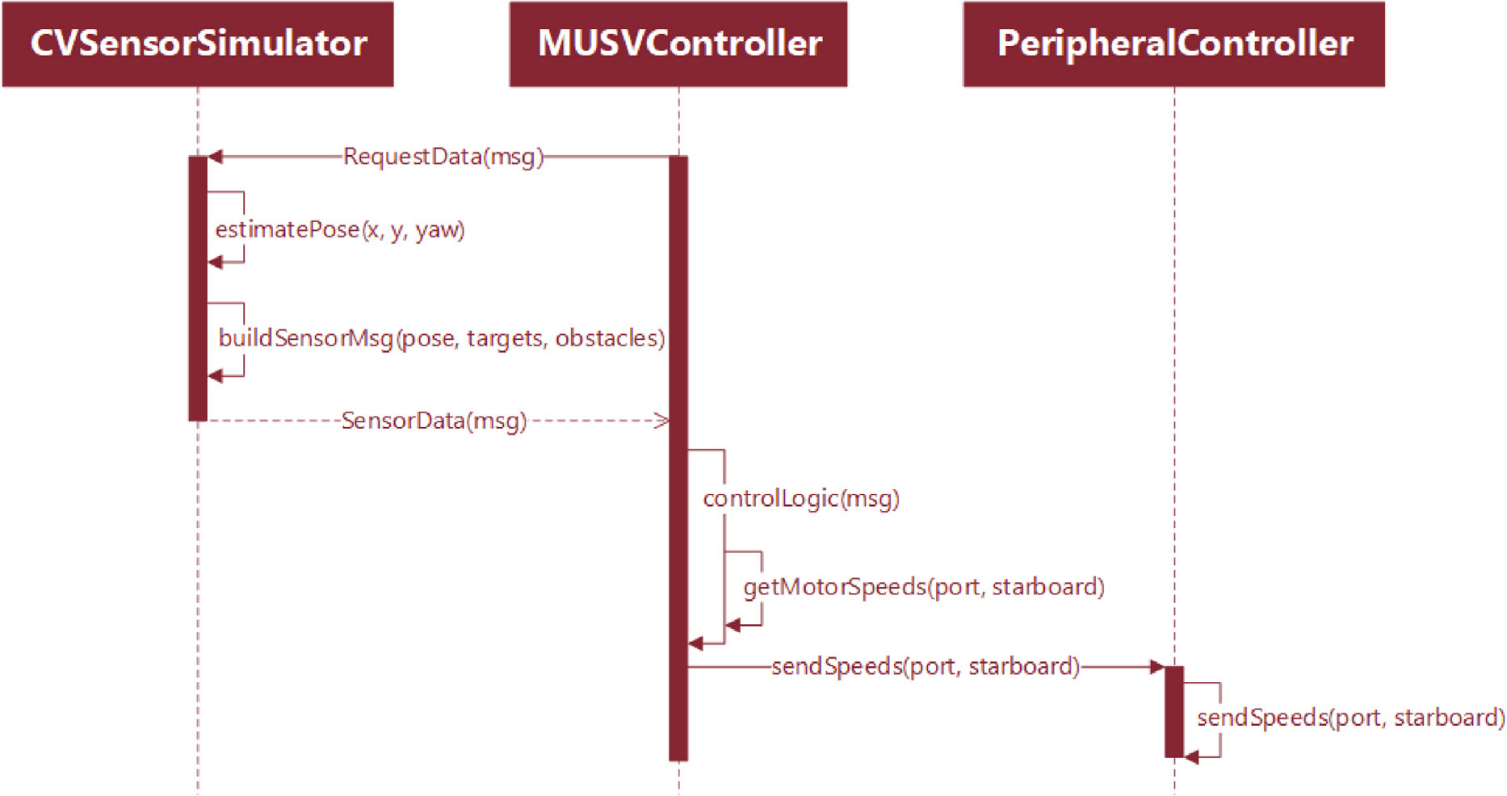
Control Software Message Sequence Diagram.

Fig. 11 shows the sequence of messages used during microUSV operation: After the systems are initialized, the vessel will query the host PC for its pose. The host PC pulls data from the its local microUSV objects which are being continuously updated with AprilTag detections and sends back a pose estimate for the querying vessel. The vessel then performs its onboard control logic to produce motor commands which are sent to the motor controller via the Arduino. This process repeats continuously to form a feedback loop.

## 3 Design files

Table 1 summarizes the design files used to fabricate or modify a microUSV. It includes the project’s source code and the required 3D printed components, all presented in STL format, along with models for an optional stand. The original CAD models, created using Solidworks, are included in the project repository as well however only one file, MicroUSV.sldasm, is included in this table due to the large number of components and subassemblies. To view and modify the full model download the ‘OSF Storage/CAD Models/Solidworks Model’ folder from the project repository [14] and open this master assembly file.

• **Hull Rev 1.6.stl**: CAD model for 3D printing the microUSV’s hull.
• **Lid Rev 1.6.stl**: CAD model for 3D printing the lid for the microUSV’s hull.
• **Keel NACA0018.stl**: CAD model for 3D printing the microUSV’s keel.
• **Electronics Bracket Rev 2.1.stl**: CAD model for 3D printing the bracket which holds the microUSV’s onboard electronics.
• **Stuffing Tube Bracket Rev 2.stl**: CAD model for 3D printing the bracket which holds the stuffing tubes in place.
• **Gearmotor Bracket Rev 2.stl**: CAD model for 3D printing the bracket which holds the motors in place.
• **Stand Base Plate.stl**: CAD model for 3D printing the base of a stand for the microUSV.
• **Stand Plate Aft.stl**: CAD model for 3D printing the rear plate of a stand for the microUSV.
• **Stand Plate Fore.stl**: CAD model for 3D printing the front plate of a stand for the microUSV.
• **MicroUSV.sldasm**: CAD model master assembly for the microUSV. Download the ‘OSF Storage/CAD Models/Solidworks Model’ folder to view and edit.
• **microUSV-SourceCode-HardwareX.zip**: The source code for the microUSV project which includes the CVSensorSimulator, MUSVController, PeripheralController, and Teleop applications. The source code can also be found at:https://github.com/CalvinGregory/microUSV/releases/tag/v1.0.
• **microUSV-Raspbian-Protobuf.zip**: Disk image for the Raspberry Pi with the microUSV software and dependencies pre-installed.

**Table 1:** Design File Table.

Design File Name	File Type	Open Source License	Location of the File		
Hull Rev 1.6.stl	STL	CERN	https://osf.io/cwfyn/		
Lid Rev 1.6.stl	STL	CERN	https://osf.io/y2q7p/		
Keel NACA0018.stl	STL	CERN	https://osf.io/7q9yp/		
Electronics Bracket Rev 2.1.stl	STL	CERN	https://osf.io/hv2wx/		
Stuffing Tube Bracket Rev 2.stl	STL	CERN	https://osf.io/z93ex/		
Gearmotor Bracket Rev 2.stl	STL	CERN	https://osf.io/ya2mx/		
Stand Base Plate.stl	STL	CERN	https://osf.io/gyext/		
Stand Plate Aft.stl	STL	CERN	https://osf.io/x43pb/		
Stand Plate Fore.stl	STL	CERN	https://osf.io/c5r7d/		
MicroUSV.sldasm	Solidworks Assembly	CERN	https://osf.io/wc7sj/		
microUSV-SourceCode-HardwareX.zip	Source Code	GPL	https://osf.io/tjpc4/		
microUSV-Raspbian-Protobuf.zip	Disk Image	GPL	https://osf.io/tpx5w/		

## 4 Bill of materials

A detailed, interactive Bill of Materials (BOM) spreadsheet can be found in the project repository [14] athttps://osf.io/7ru36/ which includes vendor links for all off-the-shelf components. A simplified BOM is included in Table 2, which summarizes the components required to produce a single microUSV. The detailed BOM spreadsheet has been structured to estimate the total production cost (in Canadian dollars) as a function of the number of microUSVs being produced. Due to the large number of items per package for some components, the cost per vessel drops significantly when producing two or more microUSVs as seen in Table 3.

**Table 2:** Generalized Bill of Materials.

Component Category	Description	Cost [$CAD]	Sources of Materials	
3D Printed Components	Custom 3D printed components including the Hull, Lid, and brackets.	90.44	3D Printer (Custom)	
Electronic Devices and Wiring	Electronic devices such as the Raspberry Pi, Arduino, and motor controller as well as the components to connect them	200.18	BuyaPi, Digikey, Pololu	
Mechanical Components and Fasteners	Screws, Nuts, Propellers, Drive Shafts, etc.	146.39	Amazon, Hobby King, McMaster-Carr	
Adhesives and Sealants	Epoxy, silicone sealant, Loctite	85.00	Amazon, Hardware Stores	
	**Total:**	**522.00**

**Table 3:** Production Cost Scaling.

Number of Vessels Produced	Total Cost [$CAD]	Cost per Vessel [$CAD]	
1	522.00	522.00	
3	1,121.64	373.88	
5	1,691.43	338.29	
10	3,173.73	317.38	

## 5 Build and operation instructions

The instructions for fabrication, assembly, and testing of the microUSV are provided in detail on the project repository Wiki page [14]. The major steps are summarized below and require an estimated two days of fabrication effort for a single user to complete with an additional three days of indirect production time also required for steps such as 3D printing and adhesive curing.

• 3D print all necessary components
• Insert a ballast plate into the keel
• Mount the keel to the bottom of the hull
• Apply epoxy coating to hull and lid
• Cut and assemble two stuffing tubes
• Thread the tips of two drive shafts
• Assemble and solder the voltage bus
• Solder header pins onto all other electronic devices
• Mount all electronic devices to the electronics bracket
• Cut and terminate eight jumper cables
• Use jumper cables to connect electronic devices
• Bore propellers and one end of each shaft coupler to accommodate a $1/8^{\prime \prime }$ drive shaft
• Mount motors, drive shafts, and stuffing tubes inside the hull
• Mount batteries and electronics bracket inside the hull
• Install desktop and onboard software
• Configure lab environment

## 6 Validation and characterization

Table 4 summarizes key properties of the microUSV.

**Table 4:** microUSV Characteristics Table.

Characteristic	Value	Unit
Mass	432	g
Length Overall	230	mm
Beam (Width)	89.2	mm
Depth (Height)	121.5	mm
Draft (Depth below waterline)	78.4	mm
Metacentric Height (GM)	9.168	mm
Maximum Righting Moment	0.0869	N*m
Angle of Vanishing Stability	None	Degrees
Maximum Speed	0.59	m/s
Roll Period	1.17	Seconds
Battery Life	4 to 8	Hours
Bandwidth Requirement	200	Byte/s

It is worth noting that the battery life range listed in Table 4 will vary based on the intensity of the use case. A battery life of 4 h was achieved under continuous operation using medium-to-high motor speeds with a fresh pair of 9 V batteries during our testing. With a more typical, intermittent motor use at 50% of maximum speed, we were easily able to achieve double that operation time.

Three test cases were used to validate the functionality of the microUSV system, the videos for which are provided in Table 5: a linear path following test, an elliptical path following test, and a multi-vehicle test. Each test has the vessel(s) operate autonomously using a simple waypoint following algorithm based on Proportional-Integral (PI) controllers. The vessels are initialized with a list of waypoints which they will steer toward sequentially with their PI controller outputting motor speed commands which aim to minimize its distance-to-waypoint error and heading-angle error. This simple controller was easy to implement and will be familiar to most potential users and so was chosen over more elaborate modern controllers.

**Table 5:** System Validation Videos.

File	Format	URL
Linear Path Test Demo Video	MP4	https://osf.io/3mcdg/
Elliptical Path Test Demo Video	MP4	https://osf.io/mt6p8/
Multi-Vehicle Test Demo Video	MP4	https://osf.io/e47ud/

### 6.1 Linear path test

**Fig. 12:**
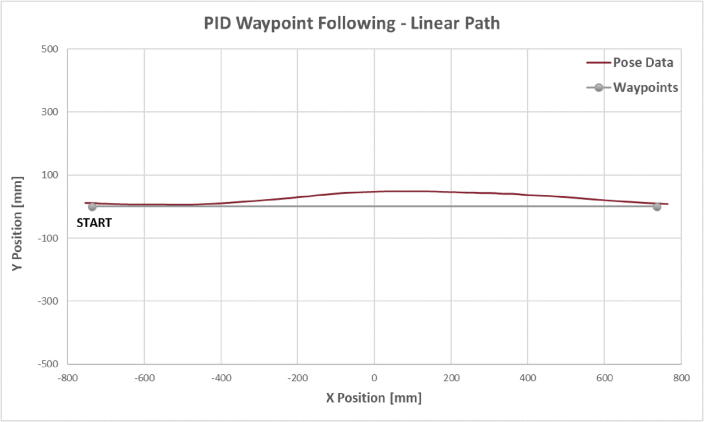
Linear Path Test Trajectory Plot.

For the linear path following test, the microUSV was placed in a test tank and instructed to travel between two waypoints positioned 1.47 m apart along the camera’s x-axis from left to right. The vessel’s trajectory can be seen in Fig. 12 along with the test’s waypoints and the expected trajectory between them.

This test demonstrates the microUSV’s ability to operate under its own power, follow a pre-defined path using onboard control logic, including fine error correction. The CVSensorSimulator system functions as intended with an average update rate of 9.5 frames per second: an acceptable update rate for navigation purposes.

The vessel follows the expected trajectory very well with some small deviations. It reached the first waypoint with a small heading error and so when its waypoint target was updated the vessel overcompensated and overshot the target heading before correcting itself to arrive on target. Better controller tuning may have improved this behavior, or it may simply be due to the limitations of this control scheme: since the controller only has access to the position of its next goal and not the subsequent ones, it does not know what heading angle will be needed after reaching its next waypoint. This creates a tendency to start the next leg of its path with a non-zero heading error. This pattern is also apparent in the elliptical path test.

Even so the vessel only deviated from the expected trajectory by a maximum of 49 mm, a small margin for a 1.47 m long path. This error was calculated as the perpendicular distance from the vessel’s position to the expected trajectory line.

### 6.2 Elliptical path test

**Fig. 13:**
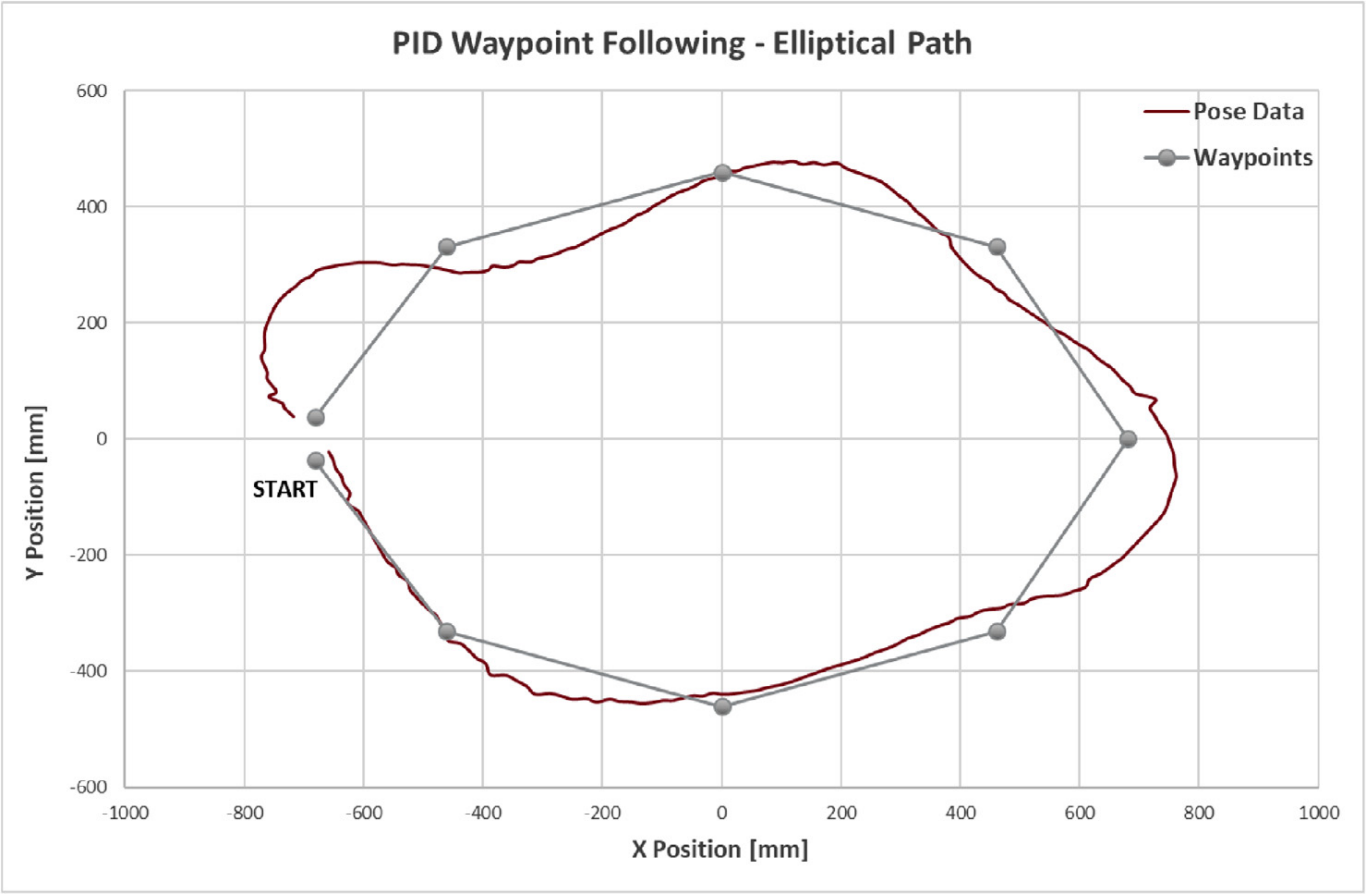
Elliptical Path Test Trajectory Plot.

The elliptical path following test uses a setup identical to that of the linear path test but with a different set of waypoints. The vessel is instructed to follow an elliptical path, or more accurately, an octagonal path whose vertices intersect an ellipse with a major diameter of 1.36 m and minor diameter of 0.92 m. The vessel starts at the left edge of the tank and proceeds through the waypoints counterclockwise. The vessel’s trajectory can be seen in Fig. 13 with the expected trajectory and waypoints shown as well.

This test demonstrates the microUSV’s ability to handle more aggressive maneuvers: It can perform tight turns without excessive roll and keeps the AprilTag in view of the overhead camera.

This trajectory deviates from its expected trajectory much more than what was observed in the linear path test, but the vessel still reaches each waypoint in sequence. Here the influence of sudden changes in heading error due to waypoint handoff is more obvious. While traveling between waypoints the vessel’s only concern is reaching the next target, so its heading is directed at the next waypoint. Once that waypoint is reached the next waypoint is considered and the vessel’s current heading now significantly off target. This leads to considerable overshoot in the vessel’s trajectory, particularly noticeable on sharp turns like that observed at the fifth waypoint on the far right of Fig. 13. The vessel still manages to achieve a reasonable approximation of the intended trajectory. Path following performance could be improved substantially by implementing a more robust control scheme such as the method described in [22].

**Fig. 14:**
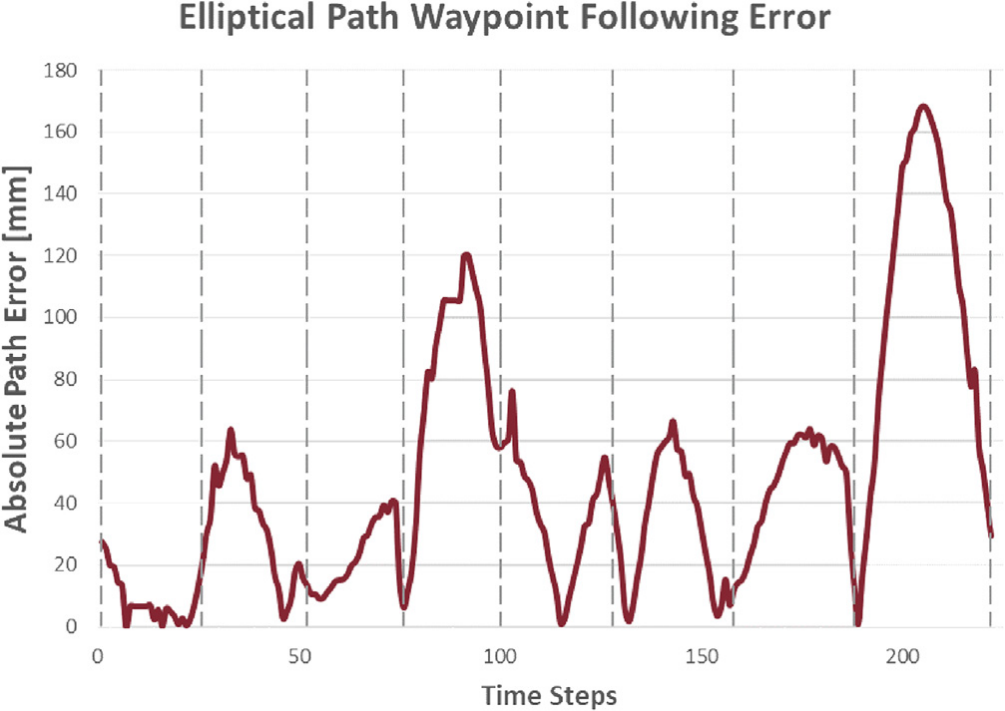
Elliptical Path Test Trajectory Error Plot.

The error, as seen in Fig. 14, was calculated as the perpendicular distance from the vessel’s position to the expected trajectory line. Due to the two-dimensional nature of the trajectory there were multiple expected trajectory line segments. The error calculation was therefore treated as a piecewise function, changing the expected trajectory line equation each time the vessel was considered to have reached its next waypoint. These transition points are denoted in Fig. 14 by the vertical dotted lines. Note how the error tends to rise sharply after reaching each waypoint before decreasing as the vessel compensates for the sudden change in goal position.

### 6.3 Multi-vehicle test

**Fig. 15:**
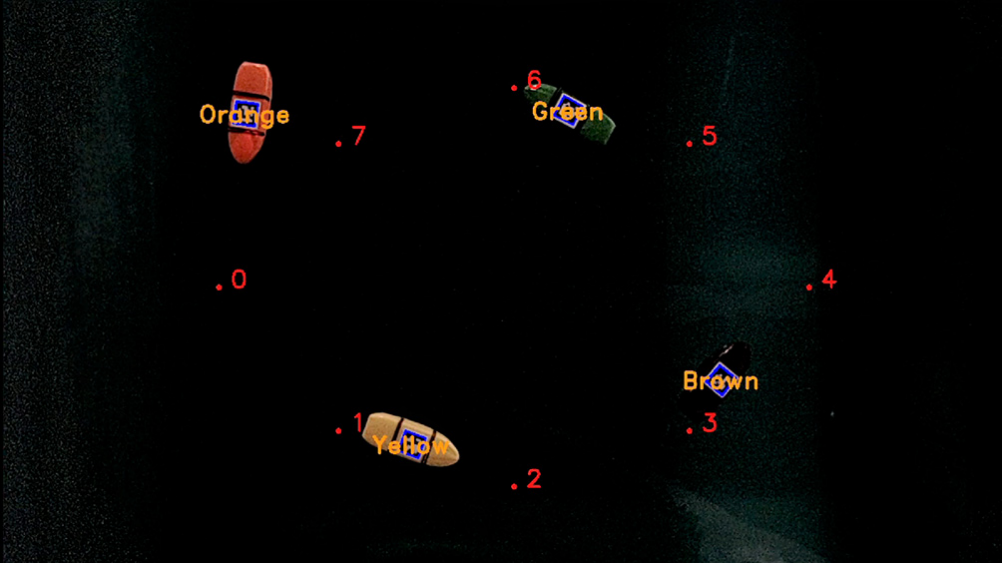
Multi-Vehicle Test Overhead Camera View.

The multi-vehicle test uses a nearly identical trajectory to the elliptical path following test where each vessel is given the same set of eight waypoints arranged in a roughly elliptical shape to follow. Unlike the elliptical test, once the vessels have reached the final waypoint in their trajectory, they are instructed to repeat that trajectory again ad infinitum. Four vessels were launched sequentially from the same position, shown in Fig. 15, with a roughly even spacing between their start times. Each vessel completes several laps of the trajectory with the first vessel successfully completing three laps of the trajectory while the fourth vessel has time for just over two laps in the 1-min, 40-s-long test with the first and last vessel launching 28 s apart.

Five and six vessel configurations were also tested and were managed by the CVSensorSimulator system without issue. Due to the limited space in the operating environment and the lack of a collision avoidance strategy in the current control scheme these tests quickly resulted in vessel collisions and pileups. These limitations will be addressed in our future work with this platform.

This test demonstrates the CVSensorSimulator system’s ability to handle the simultaneous requests of multiple vehicles with enough speed to allow for real-time navigation of each. It also shows the microUSV’s are sufficiently stable to survive the disturbances introduced by other vessels. The microUSVs appear unperturbed by the wakes created by the other vessels in the tank and, although not shown in this video, multiple inter-vehicle collisions occurred during testing, none of which resulted in a vessel overturning or the overhead camera losing view of their AprilTags.

## 7 Conclusions

This article has presented an open source USV system designed to operate in indoor laboratory environments. Built of 3D printed and off-the-shelf components, the microUSV is a cheap alternative to commercial USV systems for concept and algorithm validation in marine robotics research. The system was designed to meet a demand for low-cost marine robotics platforms suitable for swarm robotics research: a field unsatisfied by the available commercial and open source options. The system is designed to be quickly and easily modified, augmented, and reprogrammed by new users who are encouraged to find their own applications for the platform.

## Declaration of Competing Interest

The authors declare that they have no known competing financial interests or personal relationships that could have appeared to influence the work reported in this paper.

## Footnotes

1 The data shown in Fig. 10 was obtained at a later point in time using a better camera and running an updated software build. The camera used for these tests was an Intel RealSense D435, which updates faster than the Logitech C920. We recommend this new camera over the Logitech C920 to any new users going forward.
